# An Expert System Based on Fisher Score and LS-SVM for Cardiac Arrhythmia Diagnosis

**DOI:** 10.1155/2013/849674

**Published:** 2013-06-19

**Authors:** Ersen Yılmaz

**Affiliations:** Electrical-Electronic Engineering Department, Uludag University, Gorukle, 16059 Bursa, Turkey

## Abstract

An expert system having two stages is proposed for cardiac arrhythmia diagnosis. In the first stage, Fisher score is used for feature selection to reduce the feature space dimension of a data set. The second stage is classification stage in which least squares support vector machines classifier is performed by using the feature subset selected in the first stage to diagnose cardiac arrhythmia. Performance of the proposed expert system is evaluated by using an arrhythmia data set which is taken from UCI machine learning repository.

## 1. Introduction

Developing expert systems for medical diagnosis has received increasing attention in the literature for the last few decades. These systems are designed by using knowledge discovery in patients' data and machine learning algorithms. They have potentials to optimize medical decisions, improve medical treatments, and reduce financial costs [1]. 

Electrocardiogram (ECG) is graphical representation of heart's electrical activity recorded through electrodes positioned at strategic points on a body. Although it is the oldest cardiologic test, it continues as the most commonly used cardiologic test [2]. Cardiac arrhythmias are any alterations of cardiac rhythm, and they cause disruption in normal synchronized contraction sequence of heart and influence pumping efficiency. Their type and occurrence frequency make characteristic changes on ECG [3]. They are important causes of morbidity and mortality [4]. Since they can be suppressed by drugs used in treatment of arrhythmias, early recognition is important [3].

An automated system for arrhythmia analysis was first used in the early 1960s [5]. Since then, many methods have been proposed for arrhythmia diagnosis. Performances of the important group of these methods [6–10] have been evaluated on the arrhythmia data set taken from UCI machine learning repository, which is donated by Guvenir et al. [6].

In [6], an algorithm, referred to as the VFI5 (Voting Feature Intervals), was proposed for arrhythmia diagnosis. Its performance was evaluated on the arrhythmia data set by running 10-fold-cross-validation. In the same study, feature weights were learned by Genetic Algorithm (GA).

Decision Trees (DTI), Feed-Forward Neural Networks (NN), and K-Nearest Neighbors (KNN) classifiers with a variable selection algorithm based on Markov Blanket (MB), called as HITON, were applied to diagnose cardiac arrhythmia [7]. Such classifiers' performances were evaluated on the arrhythmia data set by using 10-fold-cross-validation procedure for the feature subsets determined by HITON.

Three different machine learning algorithms, namely, OneR, J48, and Naive Bayes, were used for cardiac arrhythmia diagnosis [8]. Their performances were evaluated on the arrhythmia data set by splitting it into two mutually disjoint sets as training and testing. Three different percentages of splitting were used as 50% train-50% test, 70% train-30% test, and 80% train-20% test.

In [9], an Artificial Immune Recognition System (AIRS) with Fuzzy Weighted Preprocessing (FWP) was proposed for cardiac arrhythmia diagnosis. Performance of the method was evaluated on the arrhythmia data set by using both 10-fold-cross-validation and data-splitting procedures. Like in [8], the same methodology was used for splitting the data set into training and testing sets. 

A Correlation-Based Feature Selection (CBFS) algorithm and Random Forests (RF) algorithm were used together as a diagnosing strategy for cardiac arrhythmia [10]. Strategy's performance was evaluated using the arrhythmia data set with and without random sampling by running 10-fold-cross-validation.

In this work, we propose an expert system based on Fisher Score (FS) and Least Squares Support Vector Machines (LS-SVM) for cardiac arrhythmia diagnosis. Its robustness is examined by running 10-fold-cross-validation using the arrhythmia data set taken from UCI machine learning repository. Performance of the method is evaluated in terms of classification accuracy. Additionally, confusion matrix, sensitivity, and specificity rates are presented in order to analyze the system's performance in detail. 

## 2. Fisher Score (FS)

FS [11] is one of the most widely used supervised feature selection algorithm for determining the most discriminative subset of features. It computes a score for each feature and then selects the desired number of features according to their scores. Given a data set of *N* records (*x*_*i*_, *y*_*i*_)_*i*=1_^*N*^ with *x*_*i*_ ∈ *ℜ* ^*p*^ and   *y*_*i*_ ∈ {1, 2,…, *c*}, where *x*_*i*_ is the input vector which has *p* features and *y*_*i*_ is the corresponding class label, the most discriminative subset of *m* features is determined in two sequential steps.

In the first step, FSs for all features are computed by using [12]

(1) $$\begin{array}{c} F\left(j\right)=\frac{\sum_{k=1}^{c}n_{k}{\left(\mu_{k}^{j}-\mu_{j}\right)}^{2}}{{\left(\sigma^{j}\right)}^{2}},\;j=1,2\ldots ,p, \end{array}$$

where *n*_*k*_ represents the number of records in class *k*, *μ* ^*j*^ and *σ* ^*j*^ = ∑_*k*=1_^*c*^ *n*_*k*_(*σ*_*k*_^*j*^)^2^ are the mean and the standard deviations of the entire data set corresponding to feature *j*, respectively, and *μ*_*k*_^*j*^ and *σ*_*k*_^*j*^ denote the mean and the standard deviations of class *k* corresponding to feature *j*, respectively. Then, in the second step, top *m* ranked features with high scores are selected as the most discriminative features.

## 3. Least Squares Support Vector Machines (LS-SVM)

Support Vector Machines (SVM) is a supervised learning algorithm based on the structural risk minimization principle of statistical learning theory [13]. SVM was first introduced to machine learning community by Boser et al. [14], and since then it has been successfully used for both regression and classification problems.

In classification problems, the objective of SVM is to separate data into two different classes with a maximum margin while minimizing empirical classification error. Detailed information about SVM can be found in [13–15]. 

The major drawback of SVM is its higher computational load arising from the need to solve the constrained quadratic programming problem. This drawback is overcome by LS-SVM proposed by Suykens and Vandewalle [16], which solves a set of linear equations instead of the quadratic programming problem.

Given a training data set of *N* records (*x*_*i*_, *y*_*i*_)_*i*=1_^*N*^ with *x*_*i*_ ∈ *ℜ* ^*p*^ and *y*_*i*_ ∈ ±1, where *x*_*i*_ is *p* dimensional input vector and *y*_*i*_ is the corresponding class label, LS-SVM requires minimization of the following optimization problem:

(2) $$\begin{array}{c} \underset{w,\;b,\;e}{\min}J_{\text{LS}}\left(w,e\right)=\frac{1}{2}{||w||}^{2}+\frac{1}{2}\gamma \sum_{i=1}^{N}e_{i}^{2} \end{array}$$

subject to *y*_*i*_(*w* ^*T*^ *φ*(*x*_*i*_) + *b*) = 1 − *e*_*i*_, *i* = 1 … *N*, where *w* is a parameter vector, *b* is a bias term, *e*_*i*_ is prediction error for the record *i*, *γ* is a regularization parameter, and *φ*(*x*_*i*_) is a nonlinear mapping function of the records from input space to higher dimensional feature space. The corresponding Lagrangian function for (2) is defined as follows:

(3) LLS(w,b,e;α)=JLS(w,e)−∑i=1Nαi{yi[wTφ(xi)+b]−1+ei},

where *α*_*i*_'s are Lagrange multipliers. 

According to optimality conditions ∂*L*_Ls_/∂*α*_*i*_ = 0,  ∂*L*_Ls_/∂*w* = 0, ∂*L*_Ls_/∂*b* = 0, and, ∂*L*_Ls_/∂*e*_*i*_ = 0, we can get *y*_*i*_[*w* ^*T*^ *φ*(*x*_*i*_) + *b*] − 1 + *e*_*i*_ = 0,  *w* = ∑_*i*=1_^*N*^ *α*_*i*_ *y*_*i*_ *φ*(*x*_*i*_),   ∑_*i*=1_^*N*^ *α*_*i*_ *y*_*i*_ = 0, and *α*_*i*_ = *γe*_*i*_, for *i* = 1 … *N*.

Defining *Y* = [*y*_1_; …; *y*_*N*_], $\vec{1}$ = [1; …; 1], *α* = [*α*_1_; …; *α*_*N*_], *e* = [*e*_1_; …; *e*_*N*_], and *Z* = [*φ*(*x*_1_)^*T*^ *y*_1_;…;*φ*(*x*_*N*_)^*T*^ *y*_*N*_] and after eliminating *w* and *e*, the following set of linear equations is obtained [16]:

(4) $$\begin{array}{c} \left[\frac{0\;|\;-Y^{T}}{Y\;|\;\begin{array}{c} ZZ^{T}+\gamma^{-1}I \end{array}}\right]\left[\frac{b}{\alpha }\right]=\left[\frac{0}{1}\right]. \end{array}$$

Mercer's condition can be applied to the matrix *Ω* = *ZZ* ^*T*^

(5) $$\begin{array}{c} \Omega_{i,j}=y_{i}y_{j}\varphi {\left(x_{i}\right)}^{T}\varphi \left(x_{j}\right)=y_{i}y_{j}K\left(x_{i},x_{j}\right),\;i,j=1,\ldots ,N, \end{array}$$

where *K*(*x*_*i*_, *x*_*j*_) is a kernel function representing product of two vectors in feature space, that is, *φ* ^*T*^(*x*_*i*_)*φ*(*x*_*j*_).

LS-SVM classifier is expressed as in (6) and found by solving the set of linear equations given in (4) as follows:

(6) $$\begin{array}{c} f\left(x\right)=\mathrm{sign}\left(\sum_{i=1}^{N}y_{i}\alpha_{i}K\left(x,x_{i}\right)+b\right). \end{array}$$

## 4. Arrhythmia Data

The arrhythmia data set used in this study was taken from UCI machine learning repository [6]. This data set has 452 ECG records described by 279 features, 206 features are linear, and the remaining 73 features are nominal. The first four features are age, sex, height, and weight, and the rest are features extracted from patients' ECG signals. Each record belongs to one of the 16 classes. The class 01 refers to normal ECG, the classes 02–15 refer to different arrhythmia types, and the class 16 refers to unclassified records. There are 22 unclassified records in the arrhythmia data set. Additionally, about 0.33% of the feature values in the data set are missing. Detailed description of the data set can be found on http://archive.ics.uci.edu (last accessed: April, 2013).

## 5. Performance Evaluation

The proposed expert system's performance is examined by running 10-fold-cross-validation. Four different measures, which are classification accuracy, confusion matrix, sensitivity, and specificity, are used for performance evaluation. Cross-validation procedure and these four measures are explained in the following subsections. 

### 5.1. Cross-Validation (CV)

CV is a widely used statistical method to evaluate classifiers' performances by splitting a data set into two sets as training and testing. In CV, the training and the testing sets must cross over in successive rounds, and in this way each record has a chance of being validated against [17]. For 10-fold-cross-validation, the data set is divided into 10 equal sized folds, and 10 iterations are performed. In each iteration step, one of the 10-fold is used for testing, and the remaining ninefold are used for training. In this way, at the end of the ten iteration steps, each record in the data set is used once for testing purpose.

### 5.2. Classification Accuracy

Classification accuracy is the most commonly used measure for determining performance of classifiers. It is the rate of number of correct predictions made by a model over a data set [18].

### 5.3. Confusion Matrix

Confusion matrix shows predicted and actual classifications. A confusion matrix for a classification problem with two classes is of size 2 × 2, and it is given in Table 1 [18].

### 5.4. Sensitivity and Specificity

Sensitivity is the true positive rate, and specificity is the true negative rate [18]. They are defined as in (7) and (8), respectively,

(7) $$\begin{array}{c} \text{Sensitivity}=\frac{\text{TP}}{\text{TP}+\text{FN}}, \end{array}$$

(8) $$\begin{array}{c} \text{Specificity}=\frac{\text{TN}}{\text{FP}+\text{TN}}. \end{array}$$

## 6. The Proposed Expert System

**Figure 1:**
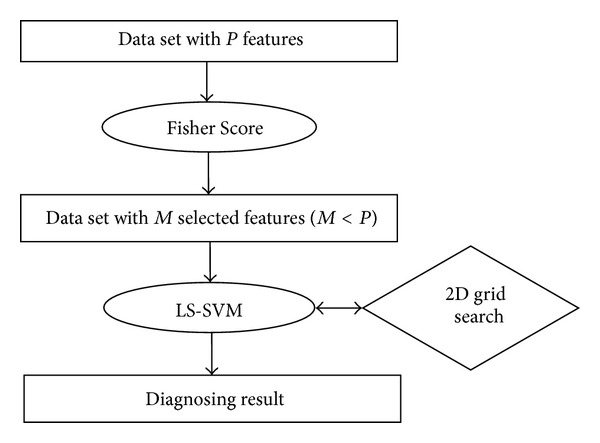
The proposed expert system's architecture.

The proposed expert system for arrhythmia diagnosis is described in this section. The system has two stages. Its architecture is given in Figure 1.

In the first stage, the feature selection algorithm FS is used to reduce the feature space dimension of the arrhythmia data set, and different sets of features are obtained. Then, in the second stage, LS-SVM classifier is performed on these feature subsets, meanwhile parameters of the classifier are optimized by using two-dimensional (2D) grid search. According to the performance results of different feature subsets, the most discriminative feature subset with the best classifier parameters are chosen, and the optimal model for expert system is created.

LS-SVM has a Gaussian kernel function given by

(9) $$\begin{array}{c} K\left(x,x_{i}\right)=\exp\left(-\frac{1}{2\sigma^{2}}{\left(x-x_{i}\right)}^{2}\right). \end{array}$$

Parameters of LS-SVM, which are penalty factor *γ* and kernel width *σ* ^2^, are optimized by using 2D grid search.

## 7. Experiments

In our experiments, unclassified records (class 16) are excluded from the data set, and the rest of the records (430 records) are grouped into two categories as presence (class 02–15) or absence (class 01) of arrhythmia. The nearest neighbour method is used to impute missing values.

The nearest neighbour is one of the most popular nonparametric missing value estimation methods. Its main advantage is its simplicity. The method uses different distance metrics to determine the similarity between the target and the reference records [19]. In this study, distances are computed by using well-known Euclidean distance. The distance from record *i* to record *j* is given by

(10) $$\begin{array}{c} d\left(x_{i},x_{j}\right)=\sqrt{{\left(x_{i}-x_{j}\right)}^{T}\left(x_{i}-x_{j}\right)},\;i,j=1,2,\ldots ,N. \end{array}$$

Missing feature values in the records are filled with the corresponding feature values of the nearest neighbour record which is selected by using (10) as the most similar record from the same class.

In the proposed expert system, LS-SVM classifier is trained nonincrementally. Therefore, the system is order independent [20, 21]. Performance evaluation of the system is made by running 10-fold-cross-validation. LS-SVM parameters (*γ*, *σ* ^2^) are selected by using 2D grid search on the intervals [0.001, 1000]. In the experiments, 55 different feature subsets and 400 different LS-SVM parameters sets for each feature subset are tested.

The experiments are performed in the following sequential steps.

**Step 1**

Different subsets of the features are obtained by the feature selection method FS.

**Step 2**

The arrhythmia data set is randomly split into 10-fold of almost equal size while maintaining the class distributions in each fold roughly the same as those in the data set.

**Step 3**

One of the feature subsets obtained in Step 1 is fed into LS-SVM.

**Step 4**

LS-SVM parameters are set to initial values, which are the first values of the selected intervals for the parameters.

**Step 5**

LS-SVM with the determined parameter values is performed by running 10-fold-cross-validation.

**Step 6**

Classification accuracies of tenfold and overall classification accuracy of these tenfold are obtained.

**Step 7**

If all values in the intervals are fed into LS-SVM, then LS-SVM parameter values with the highest overall classification accuracy are recorded for the relevant feature subset; go to Step 9, otherwise go to Step 8.

**Step 8**

New values of LS-SVM parameters are determined by 2D grid search on the intervals; go back to Step 5.

**Step 9**

If all feature subsets are fed into LS-SVM, then go to Step 11, otherwise go to Step 10.

**Step 10**

A new feature subset is fed into LS-SVM, and go back to Step 4.

**Step 11**

The feature subset with the highest overall classification accuracy is chosen as the best discriminative subset, and the relevant parameter values are used for optimum values for LS-SVM classifier.

## 8. Discussions

In our experiments, the highest overall classification accuracy is achieved when size of the feature subset is 65, and LS-SVM parameters are as follows: *γ* = 0.1 and *σ* ^2^ = 5. 

10-fold-cross-validation results of the proposed expert system with this feature subset and LS-SVM parameters are summarized in Tables 2, 3, and 4. Classification accuracies for tenfold are obtained as in Table 2.

Overall classification accuracy of the proposed system is computed by averaging the classification accuracies of tenfold, which is 82.09%.

In order to analyze the proposed expert system's performance in detail, a confusion matrix is built and sensitivity and specificity measures are computed.  Table 3 shows the confusion matrix of the proposed expert system.

Sensitivity and specificity rates of the proposed expert system are obtained as 84.86% and 80.00%, respectively.

In order to make a comparison, classification accuracies of the studies in the literature and our proposed expert system are given in Table 4. Performances of all methods given in Table 4 were evaluated on the same cardiac arrhythmia data set taken from UCI machine learning repository. 

It can be seen from the comparison table that the proposed expert system achieves a remarkable classification accuracy rate of 82.09% and it is superior to other methods except RF-CBFS with random sampling strategy. Note that the classification accuracy rate of 90% for the RF-CBFS method was achieved by randomly sampling the data set so that the class distributions were changed in the training stage.

## 9. Conclusions

In this work, an expert system based on FS and LS-SVM is proposed for cardiac arrhythmia diagnosis. A Gaussian radial basis function is used as a kernel of LS-SVM, and the parameters of LS-SVM are optimized by using 2D grid search. The proposed system's performance is evaluated using a real data set with respect to classification accuracy with 10-fold-cross-validation. Additionally, confusion matrix, sensitivity, and specificity rates are presented for further analysis of the system. 

The experiments on the arrhythmia data set show that 65 features are sufficient for the proposed expert system to perform significantly well in distinguishing among normal and arrhythmia ones, and the system achieves a remarkable classification accuracy rate of 82.09%. The sensitivity and the specificity rates are obtained as 84.86% and % 80.00, respectively. According to empirical results, it is concluded that the proposed expert systems can help clinicians make better diagnosis of cardiac arrhythmia.

## Figures and Tables

**Table 1:** Confusion matrix.

Predicted	Actual	
	Positive	Negative
Positive	TP (true positive)	FP (false positive)
Negative	FN (false negative)	TN (true negative)

TP represents an instance, which is actually positive and predicted by the model as positive.

FN represents an instance, which is actually positive but predicted by the model as negative.

TN represents an instance, which is actually negative and predicted by the model as negative.

FP represents an instance, which is actually negative but predicted by the model as positive.

**Table 2:** Classification accuracies for tenfold.

Fold-1	Fold-2	Fold-3	Fold-4	Fold-5	Fold-6	Fold-7	Fold-8	Fold-9	Fold-10
%90.69	%76.74	%72.09	%81.39	%86.05	%90.70	%81.40	%79.07	%88.37	%74.42

**Table 3:** Confusion matrix of the proposed expert system.

Predicted	Actual	
	Arrhythmia	Normal
Arrhythmia	157	49
Normal	28	196

Total	185	245

**Table 4:** Comparison of proposed expert system with the studies in the literature.

Method	Performance criteria	Maximum classification accuracy
VFI5-GA [6]	10-fold-cross-validation	68%

DTI-HITON	10-fold-cross-validation	71.87%
NN-HITON [7]		60.38%
KNN-HITON		65.30%

OneR	70% train-%30 test	58.09%
J48 [8]		74.26%
Naïve Bayes		75.00%

AIRS-FWP [9]	10-fold-cross-validation 80% train-%20 test	76.20% 80.71%

RF-CBFS [10] RF-CBFS with random sampling	10-fold-cross-validation	76.30% 90.00%

Proposed expert system (FS-LS-SVM)	10-fold-cross-validation	82.09%

## References

[1] Floares AG (2010). Using computational intelligence to develop intelligent clinical decision support systems. *Computational Intelligence Methods for Bioinformatics and Biostatistics*.

[2] Khan MG (2003). *Rapid ECG Interpretation*.

[3] Coast DA, Stern RM, Cano GG, Briller SA (1990). An approach to cardiac arrhythmia analysis using hidden Markov models. *IEEE Transactions on Biomedical Engineering*.

[4] Keating MT, Sanguinetti MC (2001). Molecular and cellular mechanisms of cardiac arrhythmias. *Cell*.

[5] Feldman CL (1983). Computer detection of cardiac arrhythmias: historical review. *American Review of Diagnostics*.

[6] Guvenir HA, Acar B, Demiroz G, Cekin A Supervised machine learning algorithm for arrhythmia analysis.

[7] Aliferis CF, Tsamardinos I, Statnikov A HITON: a novel Markov blanket algorithm for optimal variable selection.

[8] Soman T, Bobbie PO (2005). Classification of arrhythmia using machine learning techniques. *WSEAS Transactions on Computers*.

[9] Polat K, Şahan S, Güneş S (2006). A new method to medical diagnosis: artificial immune recognition system (AIRS) with fuzzy weighted pre-processing and application to ECG arrhythmia. *Expert Systems with Applications*.

[10] Özçift A (2011). Random forests ensemble classifier trained with data resampling strategy to improve cardiac arrhythmia diagnosis. *Computers in Biology and Medicine*.

[11] Bishop CM (2006). *Pattern Recognition and Machine Learning*.

[12] Gu Q, Li Z, Han J Generalized fisher score for feature selection.

[13] Vapnik VN (1995). *The Nature of Statistical Learning Theory*.

[14] Boser BE, Guyon IM, Vapnik VN Training algorithm for optimal margin classifiers.

[15] Lin Y (2002). Support vector machines and the Bayes rule in classification. *Data Mining and Knowledge Discovery*.

[16] Suykens JAK, Vandewalle J (1999). Least squares support vector machine classifiers. *Neural Processing Letters*.

[17] Refaeilzadeh P, Tang L, Liu H, Liu L, Özsu MT (2009). Cross-validation. *Encyclopedia of Data Base Systems*.

[18] Kohavi R, Provost F (1998). Glossary of terms. *Machine Learning*.

[19] Eskelson BNI, Temesgen H, Lemay V, Barrett TM, Crookston NL, Hudak AT (2009). The roles of nearest neighbor methods in imputing missing data in forest inventory and monitoring databases. *Scandinavian Journal of Forest Research*.

[20] Velert ST (2012). *Incremental learning approaches to biomedical decision problems [Ph.D. thesis]*.

[21] Rüping S Incremental learning with support vector machines.

